# Numerical Simulation of Elastic Wave Field in Viscoelastic Two-Phasic Porous Materials Based on Constant Q Fractional-Order BISQ Model

**DOI:** 10.3390/ma15031020

**Published:** 2022-01-28

**Authors:** Ning Hu, Maofa Wang, Baochun Qiu, Yuanhong Tao

**Affiliations:** 1 Marine Technology and Equipment Research Center, Hangzhou Dianzi University, Hangzhou 310018, China; hooning@hdu.edu.cn (N.H.); qiubaochun@hdu.edu.cn (B.Q.); 2 School of Sciences, Zhejiang University of Science and Technology, Hangzhou 310023, China; taoyuanhong12@126.com

**Keywords:** constant Q fractional-order derivative, viscoelastic two-phasic porous materials, BISQ model, numerical simulation, elastic wave field

## Abstract

The fractional-order differential operator describes history dependence and global correlation. In this paper, we use this trait to describe the viscoelastic characteristics of the solid skeleton of a viscoelastic two-phasic porous material. Combining Kjartansson constant Q fractional order theory with the BISQ theory, a new BISQ model is proposed to simulate elastic wave propagation in a viscoelastic two-phasic porous material. The corresponding time-domain wave propagation equations are derived, and then the elastic waves are numerically simulated in different cases. The integer-order derivatives are discretised using higher-order staggered-grid finite differences, and the fractional-order time derivatives are discretised using short-time memory central differences. Numerical simulations and analysis of the wave field characterisation in different phase boundaries, different quality factor groups, and multilayered materials containing buried bodies are carried out. The simulation results show that it is feasible to combine the constant Q fractional-order derivative theory with the BISQ theory to simulate elastic waves in viscoelastic two-phasic porous materials. The combination can better describe the viscoelastic characteristics of the viscoelastic two-phasic porous materials, which is of great significance for further understanding the propagation mechanism of elastic waves in viscoelastic two-phasic porous materials and viscoelastic two-phasic porous materials containing buried bodies. This paper provides a theoretical forward simulation for fine inversion and reconstruction of layer information and buried body structure in viscoelastic two-phasic porous materials.

## 1. Introduction

Smart material is a complex material system that integrates material and structure, intelligent processing, actuation system, control system, and sensing system. Its design and synthesis involve almost all areas of high technology disciplines. The basic material components that constitute smart materials include piezoelectric materials [1], magnetostrictive materials [2], electrostrictive materials [3], and others. The emergence of smart materials will bring human civilisation to a new level. Based on dissipative structure theory, two-phasic porous materials can intelligently dissipate elastic waves. For example, the elastic waves can be attenuated by adaptively changing physical parameters such as pore fluid viscosity, porosity, and solid skeleton viscoelasticity. Therefore, the two-phasic porous material is promising to become a kind of smart material such as magnetostrictive material and electrostrictive material.

Two-phasic porous materials are the most widely available engineering materials. The propagation of bulk and interfacial waves and the response to dynamic loading in two-phasic porous materials have been important research topics in many fields such as civil engineering [4], environmental engineering [5], energy engineering [6], exploration engineering [7], seismic engineering, and geophysics [8].

Porous materials can be viewed as a matrix skeleton composed of solid particles filled with other phase materials in the pores of the skeleton, and the fillers can move, exchange, and even undergo phase changes in the pores. The pores may be interconnected or partially connected and partially disconnected. The fluid may also be viscous or nonviscous. When a pressure gradient exists in a two-phasic porous material, there is relative motion between the fluid and the skeleton. According to the different research focus, porous material models are classified into various models such as heat transfer model [9], percolation model [10], and wave model [11].

The theory of waves in porous materials has been studied for a long time. Based on the assumption of a macroscopic isotropic continuous, neglecting thermoelastic effects and microscopic inhomogeneities, conventional elastic wave field simulation techniques generally assume that the porous materials are completely elastic in which the propagation of elastic waves satisfy the D’Alembert equation, which allows elastic waves to propagate without energy loss and waveform distortion. However, this is an ideal state, and the situation is not as such in reality. In fact, two-phasic porous materials are not completely elastic. They exhibit viscoelasticity due to the semiconnectivity of the pores, the viscosity of the skeleton, and the pore fluid. Viscoelasticity attenuates and absorbs elastic waves [12]. There are many differences between the propagation of elastic waves in the real viscoelastic materials and ideal elastic ones.

The two-phasic porous materials in nature are not perfectly elastic but viscoelastic, which means that stress and strain no longer satisfy Hooke’s law of constant relationship [13]. The elastic stiffness matrix changes with time, which means that it has memory properties. Numerical simulation of the elastic wave field can deepen our understanding of the propagation mechanism of elastic waves in the viscoelastic two-phasic porous materials, which is of great importance to guide the processing of elastic data and improve the resolution of elastic data. There are three well-known viscoelastic models: the standard linear solid model [14], the approximate constant Q model [15], and the constant Q fractional-order model [16]. Earlier scholars combined ideal springs and ideal viscous pots to simulate viscoelastic models, e.g., Maxwell model (spring and viscous pot in series), Kelvin/Kelvin-Voigt model (spring and viscous pot in parallel) [17], and others. However, the above two models have the shortcomings of overattenuation and underattenuation for high-frequency components, respectively, so that the mechanical characteristics of viscoelastic materials cannot be described realistically. Zener [18] obtained a Zener model (standard linear solid model) consisting of three mechanical elements by paralleling an ideal spring with the Maxwell model. Compared with the above two models, the Zener model can better reflect the viscoelastic behaviour of the viscoelastic materials, and I s closer to the actual situation. The quality factor Q is often used to characterise the viscoelastic materials’ absorption and attenuation of elastic wave energy. Experimental observations show that in the conventional narrow band range, the quality factor of most viscoelastic materials does not vary essentially with the frequency of the elastic wave and can be regarded as a viscoelastic material with a completely constant Q. The generalised standard linear solid model (generalised Zener model), obtained by concatenating multiple standard linear solids, can better characterise the viscoelastic materials with constant Q in any given elastic frequency band and has been widely used to describe near-constant Q viscoelastic models. Numerical simulations have been carried out using a range of methods [19,20,21]. For computational efficiency, these generalised models have been rarely used in practical elastic wave field forward simulation, imaging, and other applications. Kjartansson has developed a completely constant Q model that can quantitatively describe the absorption dispersion relations of viscoelastic, and this fully constant Q model contains fractional-order time derivatives [22,23]. Fractional calculus has received much interest in the last decades. Many applications related to the fractional-order derivatives have already been addressed in the literature, in physics [24,25], in mechanical fluids [26], in finance models [27,28,29], in diffusion equations [30], and others. It is important to mention that there exist many fractional-order derivatives, which are not cited above, derived from the Caputo derivatives and the Riemann–Liouville derivatives. The traditional Maxwell, Voigt, Kelvin, and Zener models cannot describe such complex mechanical properties accurately and concisely, but the fractional-order calculus can compensate for the shortcomings of these models [31]. Therefore, we choose to use the constant Q fractional-order model to describe the viscoelasticity of the skeleton of two-phasic materials.

In the past decades, the wave theory related to two-phasic porous materials has been supported by knowledge in multiple fields such as petrophysics, hydrodynamics, elastodynamics, anisotropy theory, and viscoelastic theory. Scholars have developed many theoretical models of porous materials. In general, the development of theoretical models of porous materials has gone from the initial fluid replacement materials to the solid–liquid coupled two-phase (multiphase) materials and then to the fracture porous materials; from the initial uniform isotropic two-phase materials to the lateral isotropic materials and then to the anisotropic two-phase materials; from the initial completely elastic materials to the viscoelastic materials. When elastic waves propagate in the porous materials, relative motion between the solid and liquid will occur due to the pressure gradients. As the porous fluid satisfies Darcy’s law, it will regain the pressure equilibrium state, during which the relative translational and rotational friction between the solid and liquid phases will convert the elastic wave energy into heat. Based on the introduction of macroscopic elastic dynamics, Biot [32,33] used Lagrange’s Equation and derived the wave equation for fluid-saturated porous applicable to the entire frequency range. In this model, the two-phase porous materials contain single uniform pores, both of which are filled with liquid. Biot introduces the dissipation function containing the relative displacement of solid and fluid phases, derives the Lagrangian equation with dissipation terms, and then derives the equation of motion. According to the principle of elastodynamics and the principle of fluid mass conservation, the pressure expression is given, and then the porous elastic Biot equation is derived. Plona [34], Kelder and Smeulders [35] observed slow P waves in artificial porous materials composed of water-saturated sintered glass and natural sandstone, respectively, confirming the correctness of the Biot theory. Biot theory can well describe the attenuation and loss of elastic waves in fluid saturated porous materials and can predict the presence of slow P waves.

When the waves propagate in two-phasic porous materials, the macroscopic Biot flow mechanism and the microscopic Squirt flow mechanism act as a coupling process to influence the propagation and attenuation of elastic waves. Dvorkin and Nur [36] started from the porous isotropic one-dimensional problem and proposed a Biot-Squirt model that treats both decay mechanisms in a unified way, taking into account the free flow of the fluid in the axial direction (reflecting Biot global flow) and the flow in the radial direction (reflecting Squirt local flow), which well explains the high dispersion and strong decay observed in the experiment. Parra [37] extended the one-dimensional isotropic BISQ model to the transversely porous isotropic case starting from the frequency-domain porous elastic wave equation. Yang and Zhang [38] extend it to the high-dimensional transversely isotropic and generally anisotropic case to the high-dimensional transverse isotropic and general anisotropic case. Many scholars have further refined and developed the basic theory and application of the BISQ model.

The BISQ model can well describe the relative motion between flow–solid phases in two-phasic porous materials, including macroscopic Biot flow and microscopic Squirt flow. However, this is only one factor leading to the attenuation of elastic waves. The viscoelasticity of the solid skeleton of the two-phasic porous material also cannot be negligible. We use fractional-order derivatives to describe the viscoelasticity of the solid skeleton and innovatively introduce them into the BISQ model to establish a new fractional-order viscoelastic BISQ model. The two main factors leading to the attenuation of elastic waves, namely the viscoelasticity of the solid skeleton and the relative flow between solid and liquid, are considered. This will be closer to the actual state than the results of previous studies. Through the numerical simulation of elastic wave propagation in viscoelastic two-phasic porous materials, we can explore the mechanism of elastic wave propagation in viscoelastic two-phasic porous materials, which helps us analyse and study the propagation and response law and characteristics of elastic waves in viscoelastic two-phasic porous materials. This new fractional BISQ model can be applied to many fields in the future. Subsurface reservoirs can be regarded as fluid-saturated two-phasic porous materials. Seismic wave simulation is the most fundamental problem in seismic exploration. Establishing a seismic wave propagation model that more closely resembles the actual subsurface medium and obtaining wavefield solutions by numerical methods are important steps in illuminating the subsurface using seismic imaging and waveform inversion. Seafloor sediments can also be regarded as a kind of two-phasic porous medium. Based on the propagation mechanism of acoustic waves in the two-phasic porous medium of the seafloor, the accepted acoustic data can be used to invert the seafloor sediment parameters, which is a more important aspect in the field of marine geophysics. Underwater anechoic materials can also be two-phasic porous materials. Therefore, the study of elastic wave propagation mechanisms in two-phasic porous materials is also crucial for designing underwater anechoic materials or even intelligent anechoic materials.

## 2. Methods

### 2.1. Constant Q Fractional-Order Constitutive Relationship

In terms of the stress–strain constitutive relationship, the linear viscoelasticity of the material skeleton can be expressed as the convolution of the relaxation function with the strain on the time derivative [39],

(1) $$\sigma_{ij}\left(X,t\right)=C_{ijkl}\left(t\right)*\partial_{t}\varepsilon_{kl}\left(X,t\right)$$

where ∗ denotes the convolution operation, X denotes the spatial location, t represents the time variable, $C_{ijkl}\left(t\right)$ is a fourth-order relaxation tensor function, and $\sigma_{ij}\left(X,t\right)$ and $\varepsilon_{kl}\left(X,t\right)$ are the stress tensor and strain tensor, respectively.

For an isotropic solid skeletal, the fourth-order relaxation tensor of 81 components degenerates into a viscoelastic material stiffness matrix, which requires only two independent relaxation tensor representations.

(2) $$C\left(t\right)=\left[\begin{array}{cccccc} C_{11}\left(t\right) & C_{12}\left(t\right) & C_{12}\left(t\right) & & & \\ C_{12}\left(t\right) & C_{11}\left(t\right) & C_{12}\left(t\right) & & & \\ C_{12}\left(t\right) & C_{12}\left(t\right) & C_{11}\left(t\right) & & & \\ & & & C_{44}\left(t\right) & & \\ & & & & C_{44}\left(t\right) & \\ & & & & & C_{44}\left(t\right) \end{array}\right]$$

where $c_{12}\left(t\right)=c_{11}\left(t\right)-2c_{44}\left(t\right)$, $c_{11}\left(t\right)$ and $c_{44}\left(t\right)$ are two independent relaxation functions, $c_{44}\left(t\right)=2\mu \left(t\right)$ and $c_{11}\left(t\right)=\lambda \left(t\right)+2\mu \left(t\right)$, where λ and μ are the Larmé constants for isotropic materials.

For a transversely isotropic (VTI), only five independent relaxation tensor components are required to describe the stiffness matrix of viscoelastic materials.

(3) $$C\left(t\right)=\left[\begin{array}{cccccc} C_{11}\left(t\right) & C_{12}\left(t\right) & C_{13}\left(t\right) & & & \\ C_{12}\left(t\right) & C_{11}\left(t\right) & C_{13}\left(t\right) & & & \\ C_{13}\left(t\right) & C_{13}\left(t\right) & C_{33}\left(t\right) & & & \\ & & & C_{44}\left(t\right) & & \\ & & & & C_{44}\left(t\right) & \\ & & & & & C_{66}\left(t\right) \end{array}\right]$$

where $c_{12}\left(t\right)=c_{11}\left(t\right)-2c_{66}\left(t\right)$, $c_{11}\left(t\right)$ and $c_{33}\left(t\right)$ are two independent relaxation functions corresponding to the horizontal and vertical components of the P-wave modulus, respectively. $c_{11}\left(t\right)=\lambda_{∥}\left(t\right)+2\mu_{∥}\left(t\right)$, $c_{33}\left(t\right)=\lambda_{⊥}\left(t\right)+2\mu_{⊥}\left(t\right)$, where $\lambda_{∥}$, $\mu_{∥}$ and $\lambda_{⊥}$, $\mu_{⊥}$ are the Lamé constants for VTI materials parallel and perpendicular to the formation, respectively. $c_{13}\left(t\right)=\lambda_{⊥}\left(t\right)$, the shear modulus $c_{44}\left(t\right)=\mu_{*}\left(t\right)$, $c_{66}\left(t\right)=\mu_{∥}\left(t\right)$, where $\mu_{*}\left(t\right)$ is the shear modulus connecting the two directions. This paper mainly focuses on transversely isotropic (VTI) materials. Substituting Equation (3) into Equation (1), in the x−z plane we have

(4) $$\{\begin{array}{c} \sigma_{xx}=C_{11}\left(t\right)*\partial_{t}\varepsilon_{xx}+C_{13}\left(t\right)*\partial_{t}\varepsilon_{zz}\\ \sigma_{zz}=C_{13}\left(t\right)*\partial_{t}\varepsilon_{xx}+C_{33}\left(t\right)*\partial_{t}\varepsilon_{zz}\\ \sigma_{xz}=C_{44}\left(t\right)*\partial_{t}\varepsilon_{xz} \end{array}$$

where

$$C_{11}\left(t\right)=\frac{M_{p∥}}{\Gamma \left(1-2\gamma_{p}\right)}{\left(\frac{t}{t_{0}}\right)}^{-2\gamma_{p}}H\left(t\right)$$

$$C_{13}\left(t\right)=\frac{M_{13}}{\Gamma \left(1-2\gamma_{13}\right)}{\left(\frac{t}{t_{0}}\right)}^{-2\gamma_{13}}H\left(t\right)$$

$$C_{33}\left(t\right)=\frac{M_{Ρ⊥}}{\Gamma \left(1-2\gamma_{p}\right)}{\left(\frac{t}{t_{0}}\right)}^{-2\gamma_{p}}H\left(t\right)$$

$$C_{12}\left(t\right)=\frac{M_{12}}{\Gamma \left(1-2\gamma_{12}\right)}{\left(\frac{t}{t_{0}}\right)}^{-2\gamma_{12}}H\left(t\right)$$

$$C_{44}\left(t\right)=\frac{M_{\mu }}{\Gamma \left(1-2\gamma_{s}\right)}{\left(\frac{t}{t_{0}}\right)}^{-2\gamma_{s}}H\left(t\right)$$

H(t) is the Heaviside step function, and $\omega_{0}=1/t_{0}$ is the reference frequency.

According to the theory of fractional-order derivatives [40], the convolution operation in Equation (4) is transformed into a fractional-order time derivative, and then Equation (4) becomes

(5) $$\{\begin{array}{c} \sigma_{xx}=C_{p∥}D^{2\gamma_{p}}\varepsilon_{xx}+\tilde{C_{13}}D^{2\gamma_{13}}\varepsilon_{zz}\\ \sigma_{zz}=\tilde{C_{13}}D^{2\gamma_{13}}\varepsilon_{xx}+C_{Ρ⊥}D^{2\gamma_{p}}\varepsilon_{zz}\\ \sigma_{zx}=C_{\mu }D^{2\gamma_{s}}\varepsilon_{zx}+C_{\mu }D^{2\gamma_{s}}\varepsilon_{xz} \end{array}$$

where $D^{2\gamma }=\frac{\partial^{2\gamma }}{\partial t^{2\gamma }}$ is the time fractional-order derivative of 2γ order, and we have

$$C_{p∥}=M_{p∥}\omega_{r}{}^{-2\gamma_{p}}$$

$$C_{p⊥}=M_{p⊥}\omega_{r}{}^{-2\gamma_{p}}$$

$$C_{\mu }=M_{\mu }\omega_{r}{}^{-2\gamma_{S}}$$

$$\tilde{C_{13}}=M_{13}\omega_{r}{}^{-2\gamma_{13}}$$

where the order of the fractional-order derivative is defined as follows

$$\gamma_{p,s}=\frac{1}{\pi }tan^{-1}\left(\frac{1}{Q_{p,s}}\right)$$

$$\gamma_{12,13}=\frac{1}{\pi }tan^{-1}\left(\frac{1}{Q_{12,13}}\right)$$

where $Q_{p}$ and $Q_{s}$ are the quality factors of P- and S-wave, respectively, and $Q_{12}$, $Q_{13}$ can be expressed respectively as [41].

$$Q_{12}=Q_{P}\frac{C_{11}^{0}-2C_{44}^{0}}{C_{11}^{0}-2C_{44}^{0}\frac{Q_{P}}{Q_{S}}}$$

$$Q_{13}=Q_{P}\frac{C_{33}^{0}-2C_{44}^{0}}{C_{33}^{0}-2C_{44}^{0}\frac{Q_{P}}{Q_{S}}}$$

In addition, the reference modulus $M_{p∥}$, $M_{p⊥}$, $M_{13}$, $M_{12}$, and $M_{\mu }$ can be expressed by the P-wave velocities $v_{p∥}$, $v_{p⊥}$ and S-wave velocities $v_{s}$ at the reference frequency $\omega_{\gamma }$, respectively, as follows

$$M_{p∥}=\rho_{s}v_{p∥}^{2}{cos}^{2}\left(\frac{\pi \gamma_{p}}{2}\right)$$

$$M_{p⊥}=\rho_{s}v_{p⊥}^{2}{cos}^{2}\left(\frac{\pi \gamma_{p}}{2}\right)$$

$$M_{\mu }=\rho_{s}v_{s}^{2}{cos}^{2}\left(\frac{\pi \gamma_{S}}{2}\right)$$

$$M_{12}=C_{12}^{0}{cos}^{2}\left(\frac{\pi \gamma_{12}}{2}\right)$$

$$M_{13}=C_{13}^{0}{cos}^{2}\left(\frac{\pi \gamma_{13}}{2}\right)$$

where $\rho_{s}$ is the density of the solid skeleton, $C_{11}^{0}$, $C_{12}^{0}$, $C_{13}^{0}$, $C_{33}^{0}$, $C_{44}^{0}$, $C_{66}^{0}$ are the elements of $C^{0}$ which is the elastic stiffness matrix tensor of the dry solid skeleton. For the VTI materials, we have

$$C^{0}=\left[\begin{array}{cccccc} C_{11}^{0} & C_{12}^{0} & C_{13}^{0} & & & \\ C_{12}^{0} & C_{11}^{0} & C_{23}^{0} & & & \\ C_{13}^{0} & C_{23}^{0} & C_{33}^{0} & & & \\ & & & C_{44}^{0} & & \\ & & & & C_{44}^{0} & \\ & & & & & C_{66}^{0} \end{array}\right]$$

### 2.2. Constant Q Wave Propagation Equations

In this section, Kjartansson constant Q fractional-order viscoelastic constitutive relationship is used to describe the viscoelasticity of the solid skeleton. The viscoelastic skeleton constitutive relationship, geometric equations, equations of motion, and the expression of fluid pressure from the BISQ theory are given for two-phase VTI materials. Based on these equations, we derive the two-dimensional wave propagation equations.

According to [39], the viscoelastic skeleton constitutive relationship is

(6) $$\tau =C\left(t\right)*\partial_{t}\varepsilon \left(t\right)-\alpha \left(t\right)P\left(t\right)\;$$

where τ is the total stress tensor and ε(t) is the solid skeleton strain tensor, and α(t) is the poroelastic coefficient tensor of the effective stress.

For VTI, α(t) is

$$\{\begin{array}{c} \alpha_{11}\left(t\right)=\alpha_{22}\left(t\right)=1-\frac{C_{11}\left(t\right)+C_{12}\left(t\right)+C_{13}\left(t\right)}{3K_{s}}\\ \alpha_{33}\left(t\right)=1-\frac{2C_{13}\left(t\right)+C_{33}\left(t\right)}{3K_{s}}\\ \alpha_{ij}\left(t\right)=0,\;\;i\neq j,\;\;i,j=1,2,3 \end{array}$$

For isotropic, α(t) is

$$\{\begin{array}{c} \alpha_{11}\left(t\right)=\alpha_{22}\left(t\right)=\alpha_{33}\left(t\right)=1-\frac{C_{11}\left(t\right)+2C_{12}\left(t\right)}{3K_{s}}\\ \alpha_{ij}\left(t\right)=0,\;\;i\neq j,\;\;i,j=1,2,3 \end{array}$$

The expression of fluid pressure from the BISQ theory is

(7) $$P\left(t\right)=-\nabla \cdot \left(F\left(t\right)SU\right)-\left(F\left(t\right)S\frac{\alpha \left(t\right)-\phi I}{\phi }\right)\cdot \varepsilon \left(t\right)$$

where

$$F\left(t\right)={\left(\frac{1}{K_{f}}+\frac{1}{\phi Q_{j}\left(t\right)}\right)}^{-1}$$

is the Biot flow tensor, $K_{f}$ is the bulk modulus of the porous fluid, S is the Squirt tensor, U denotes the displacements of the fluid, ϕ is the material’s porosity, I is the unit matrix. $Q_{j}\left(t\right)$ is an intermediate variable related to the bulk modulus

$$\frac{1}{Q_{j}\left(t\right)}=\frac{\sum_{i=1}^{3}\alpha_{ij}\left(t\right)-\phi }{K_{s}},i,j=1,2,3$$

The geometric equation is

(8) $$\varepsilon_{ij}=\frac{1}{2}\left(\frac{\partial u_{i}}{\partial x_{j}}+\frac{\partial u_{j}}{\partial x_{i}}\right),\;i,j=1,2,3$$

The differential equations of motion are

(9) $$\{\begin{array}{c} \overset{3}{\underset{j=1}{\sum }}\frac{\partial \tau_{ij}}{\partial x_{j}}=\frac{\partial^{2}}{\partial t^{2}}\left(\rho_{1}u_{i}+\rho_{2}U_{i}\right)\\ -\frac{\partial }{\partial x_{i}}\left(\phi P\left(t\right)\right)=\frac{\partial^{2}}{\partial t^{2}}\left(\rho_{12i}u_{i}+\rho_{22i}U_{i}\right)+\eta \phi^{2}\overset{3}{\underset{j=1}{\sum }}r_{ij}\frac{\partial }{\partial t}\left(U_{j}-u_{j}\right) \end{array}$$

where $\tau_{ij}$ is the component of the total stress tensor, $\rho_{1}=\left(1-\phi \right)\rho_{s}$, $\rho_{2}=\phi \rho_{f}$, $\rho_{s}$ is the density of the solid phase, $\rho_{22i}=\rho_{2}-\rho_{12i}$, $\rho_{12i}=-\rho_{ai}$, $\rho_{ai}$ is the additional mass density of the solid–fluid coupling in the $x_{i}$ direction, $u_{i}$ and $U_{i}$ denote the displacements of the solid and the fluid in the $x_{i}$ direction and $x_{i}$, i=1,2,3 represent the x, y, z direction respectively, η is the fluid viscosity coefficient. Fluid impedance coefficient $r_{ij}={\left(k_{ij}\right)}^{-1}$, where $k_{ij}$ is the element of permeability tensor k, for VTI $k=diag\left(k_{11},k_{11},k_{33}\right)$.

Combining Equations (6)–(9), the wave propagation equations in VTI viscoelastic two-phase porous materials based on the fractional-order BISQ model can be derived. In the x-z plane, the wave propagation equations consist of the following five equations

$$c_{11}^{0}\left(t\right)*\frac{\partial^{3}u_{1}}{\partial t\partial x^{2}}+c_{44}^{0}\left(t\right)*\frac{\partial^{3}u_{1}}{\partial t\partial z^{2}}+\left[c_{13}^{0}\left(t\right)+c_{44}^{0}\left(t\right)\right]*\frac{\partial^{3}u_{3}}{\partial t\partial x\partial z}-\alpha_{11}\left(t\right)\frac{\partial P\left(t\right)}{\partial x}=\frac{\partial^{2}}{\partial t^{2}}\left(\rho_{1}u_{1}+\rho_{2}U_{1}\right)$$

$$\left[c_{13}^{0}\left(t\right)+c_{44}^{0}\left(t\right)\right]*\frac{\partial^{3}u_{1}}{\partial t\partial x\partial z}+\left(c_{44}^{0}\left(t\right)*\frac{\partial^{3}u_{3}}{\partial t\partial x^{2}}+c_{33}^{0}\left(t\right)*\frac{\partial^{3}u_{3}}{\partial t\partial z^{2}}\right)-\alpha_{33}\left(t\right)\frac{\partial P\left(t\right)}{\partial z}=\frac{\partial^{2}}{\partial t^{2}}\left(\rho_{1}u_{3}+\rho_{2}U_{3}\right)$$

$$-\frac{\partial }{\partial z}\left(\phi P\left(t\right)\right)=\frac{\partial^{2}}{\partial t^{2}}\left[-\rho_{ax}u_{1}+\left(\rho_{2}+\rho_{ax}\right)U_{1}\right]+\frac{\eta \phi^{2}}{k_{11}}\frac{\partial }{\partial t}\left(U_{1}-u_{1}\right)$$

$$-\frac{\partial }{\partial z}\left(\phi P\left(t\right)\right)=\frac{\partial^{2}}{\partial t^{2}}\left[-\rho_{az}u_{3}+\left(\rho_{2}+\rho_{az}\right)U_{3}\right]+\frac{\eta \phi^{2}}{k_{33}}\frac{\partial }{\partial t}\left(U_{3}-u_{3}\right)$$

$$P\left(t\right)=-\nabla \cdot \left(F\left(t\right)SU\right)-\left(F\left(t\right)S\frac{\alpha \left(t\right)-\phi I}{\phi }\right)\cdot \varepsilon \left(t\right)$$

According to Equation (5), the above equations can be further derived and can be written as the velocity-stress equations

$$\frac{\partial v_{1}}{\partial t}=A_{11}\rho_{2}\left[\frac{\partial P\left(t\right)}{\partial x}+\frac{m_{11}\phi }{\rho_{2}}\left(\frac{\partial \sigma_{11}}{\partial x}+\frac{\partial \sigma_{13}}{\partial z}\right)+\frac{\eta \phi }{k_{11}}\left(V_{1}-v_{1}\right)\right]$$

$$\frac{\partial V_{1}}{\partial t}=-A_{11}\rho_{1}\left[\frac{\partial P\left(t\right)}{\partial x}+\frac{\rho_{f}-m_{11}\phi }{\rho_{1}}\left(\frac{\partial \sigma_{11}}{\partial x}+\frac{\partial \sigma_{13}}{\partial z}\right)+\frac{\eta \phi }{k_{11}}\left(V_{1}-v_{1}\right)\right]$$

$$\frac{\partial v_{3}}{\partial t}=A_{33}\rho_{2}\left[\frac{\partial P\left(t\right)}{\partial z}+\frac{m_{33}\phi }{\rho_{2}}\left(\frac{\partial \sigma_{13}}{\partial x}+\frac{\partial \sigma_{33}}{\partial z}\right)+\frac{\eta \phi }{k_{33}}\left(V_{3}-v_{3}\right)\right]$$

$$\frac{\partial V_{3}}{\partial t}=-A_{33}\rho_{1}\left[\frac{\partial P\left(t\right)}{\partial x}+\frac{\rho_{f}-m_{33}\phi }{\rho_{1}}\left(\frac{\partial \sigma_{13}}{\partial x}+\frac{\partial \sigma_{33}}{\partial z}\right)+\frac{\eta \phi }{k_{33}}\left(V_{3}-v_{3}\right)\right]$$

$$-\frac{\partial P\left(t\right)}{\partial t}=F_{1}\left(t\right)S_{1}\frac{\partial V_{1}}{\partial x}+F_{3}\left(t\right)S_{3}\frac{\partial V_{3}}{\partial z}+F_{1}\left(t\right)S_{1}\frac{\alpha_{11}\left(t\right)-\phi }{\phi }\frac{\partial v_{1}}{\partial x}+F_{3}\left(t\right)S_{3}\frac{\alpha_{33}\left(t\right)-\phi }{\phi }\frac{\partial v_{3}}{\partial z}$$

$$\frac{\partial \sigma_{11}}{\partial t}=C_{Ρ}D^{2\gamma_{Ρ}}\frac{\partial v_{1}}{\partial x}+C_{13}D^{2\gamma_{13}}\frac{\partial v_{3}}{\partial z}-\alpha_{11}\left(t\right)\frac{\partial P\left(t\right)}{\partial t}$$

$$\frac{\partial \sigma_{33}}{\partial t}=C_{13}D^{2\gamma_{13}}\frac{\partial v_{1}}{\partial x}+C_{Ρ}D^{2\gamma_{Ρ}}\frac{\partial v_{3}}{\partial z}-\alpha_{33}\left(t\right)\frac{\partial P\left(t\right)}{\partial t}$$

$$\frac{\partial \sigma_{31}}{\partial t}=C_{\mu }D^{2\gamma_{s}}\frac{\partial v_{1}}{\partial z}+C_{\mu }D^{2\gamma_{s}}\frac{\partial v_{3}}{\partial x}$$

where

$$m_{ii}=\frac{\phi \rho_{f}+\rho_{ai}}{\phi^{2}},\;A_{ii}={\left[\rho_{1}m_{ii}\phi -\rho_{2}\left(\rho_{f}-m_{ii}\phi \right)\right]}^{-1}\;,\;i=1,3$$

### 2.3. Finite-Difference Numerical Solution

The discretisation form of the finite-difference algorithm is intuitively simple, the mesh division form is more flexible, the computational consumption is relatively small, and it is more suitable for the simulation of wave equations of complex models. The staggered-grid finite-difference algorithm is a more advanced meshing method. Compared with the conventional finite-difference meshing method, the computational accuracy is doubled without increasing the computational consumption. In this paper, the wave propagation equations established for viscoelastic two-phasic contain both integer-order derivatives and fractional-order derivatives. The integer-order derivatives are discretised using the staggered-grid finite-difference algorithm, while the fractional-order derivatives are discretised using the central difference method.

Fractional-order differential operators can describe complex mechanical and physical processes with history dependence and spatial full-domain correlation simply and accurately. One of the main mathematical features of fractional-order derivatives is their nonlocal nature, i.e., the current state is related to all past states, and thus the fractional-order wave equations require an enormous amount of computation and storage in numerical simulations, especially for long time histories or sizeable computational domain. In order to reduce the cost of computation and improve computational efficiency, Podlubny [42] proposed the “short-time memory algorithm”, which truncates the length of the operator to achieve the goal of reducing the amount of operations and storage. Only considering the finite number of operations before the current time t and the finite time interval [t−L, t] that has a large impact on the current moment, L is called the memory length.

#### 2.3.1. Discretisation of Fractional Order Time Derivatives

For fractional-order derivatives, there are various definitions, and in this paper, we use the Grünwald-Letnikov definition

(10) $$D^{2\gamma }\frac{\partial v\left(t\right)}{\partial x}=\underset{\tau \to 0}{lim}\tau^{-2\gamma }\sum_{m=0}^{\frac{t}{\tau }}\frac{{\left(-1\right)}^{m}\Gamma \left(2\gamma +1\right)}{m!\Gamma \left(2\gamma +1-m\right)}\frac{\partial v\left(t-m\tau \right)}{\partial x}$$

Define

$$\varphi \left(2\gamma ,m\right)=\frac{{\left(-1\right)}^{m}\Gamma \left(2\gamma +1\right)}{\Gamma \left(m+1\right)\Gamma \left(2\gamma +1-m\right)}$$

φ(2γ,m) satisfies the recurrence relation

$$\varphi \left(2\gamma ,m\right)=-\varphi \left(2\gamma ,m-1\right)\frac{1+2\gamma -m}{m}$$

when m=0, φ(2γ,0)=1. Using the short-time memory centre difference approximation to Equation (10). When τ→Δt, the discretisation at t=mΔt gives

$$D^{2\gamma }\frac{\partial v\left(t\right)}{\partial x}=\underset{\tau \to 0}{lim}\tau^{-2\gamma }\overset{L}{\underset{m=0}{\sum }}\varphi \left(2\gamma ,m\right)\frac{\partial v\left(t-m\tau \right)}{\partial x}$$

$$=\frac{\Delta t^{-2\gamma }}{\Delta x}\sum_{m=0}^{L}\varphi \left(2\gamma ,m\right)\sum_{n=1}^{N}a_{n}\left(v_{j+n,l}^{k-m}-v_{j-\left(n-1\right),l}^{k-m}\right)$$

#### 2.3.2. Stability and Absorption Boundary Conditions

For finite-difference numerical simulations, stability should be considered in the calculation process. The stability condition of the fractional-order partial differential equations established in this paper is obtained by Fourier stability analysis. Its expression is

$$\Delta tv\sqrt{\frac{1}{\Delta x^{2}}+\frac{1}{\Delta z^{2}}}\leq \frac{\delta }{\sum_{n=1}^{N}\left|c_{n}\right|}$$

where Δx and Δz are the horizontal and vertical spatial grid steps, Δt is the time interval, δ=0.872 is the stability constant, and $c_{n}$ is the corresponding staggered-grid difference operator coefficient.

In order to eliminate the false reflection of the artificial boundary, the practical and straightforward Cerjan decay boundary [43] is used, i.e., expanding N grid points outward along the artificial boundary

$$G=exp\left[-a{\left(N-i\right)}^{2}\right],\;1\leq i\leq N$$

where i is the node number within the absorber layer, and a is the attenuation coefficient. The optimal value of the attenuation coefficient can be determined by several tests.

## 3. Results and Discussion

The subsurface medium can be regarded as a fluid-saturated two-phasic porous material, and seismic waves belong to elastic waves. The discussion of the factors affecting the attenuation of elastic waves in a subsurface medium and the phenomena such as scattering and transmission when encountering buried bodies are representative. Therefore, to examine the effectiveness of the Constant Q fractional-order BISQ model proposed in this paper, we select different subsurface media models for the numerical simulation study of wave fields. The grid size of the simulation area is 300×300, where the spatial interval is 10 m. The source of the vibration is the Ricker wavelet, which is located at  (150,150), loading in the x-direction. The time sampling interval is set to 0.5 ms.

### 3.1. Single Layer Model with Different Phase Boundaries

The composition of the viscoelastic two-phasic porous materials is different, and the viscosity of the fluid contained in it may also be different. In order to analyse the influence of fluid viscosity coefficient on elastic wave propagation characteristics in detail, the single-layer models with ideal phase boundary and different fluid viscosity coefficients are designed. Some basic parameters are shown in Table 1.

**Figure 1:**
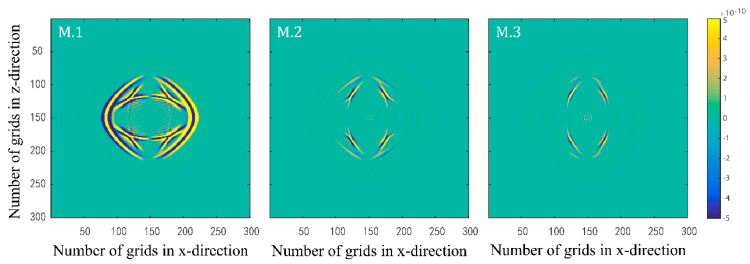
Snapshots of solid–phase x–direction velocity components with three different fluid viscosity coefficients at t=0.15 s.

**Figure 2:**
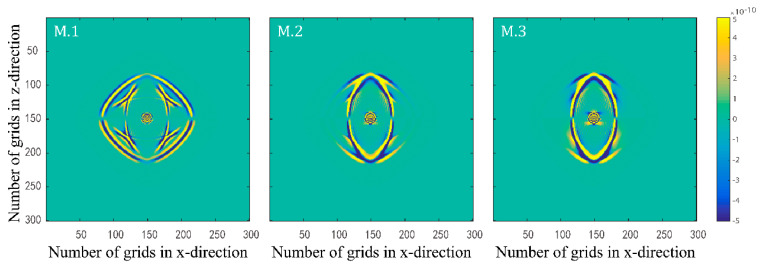
Snapshots of liquid phase z–direction velocity components with three different fluid viscosity coefficients at t=0.15 s.

**Figure 3:**
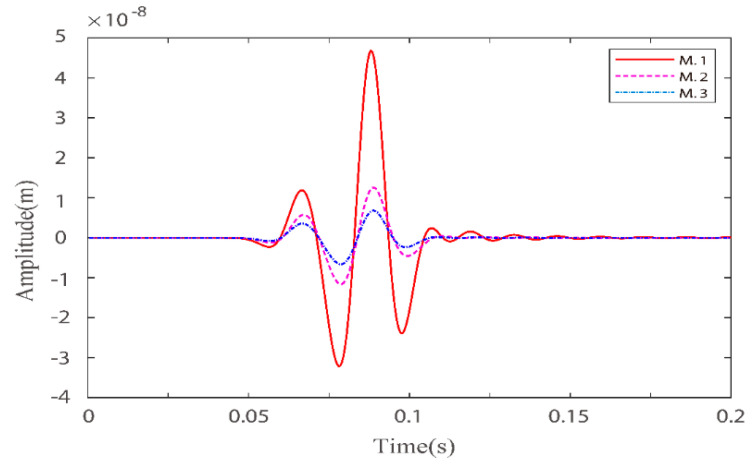
Comparison of solid–phase x–direction velocity components with three different fluid viscosity coefficients at points (160,160).

**Figure 4:**
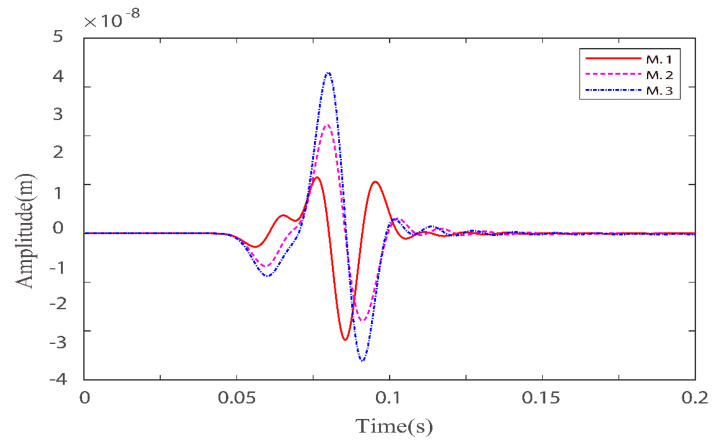
Comparison of liquid phase z–direction velocity components with three fluid viscosity coefficients at point (160,160).

Numerical simulations are performed for the three models in Table 1. Figure 1 and Figure 2 illustrate the effects of fluid viscosity coefficient on the solid-phase and liquid-phase wave fields, respectively. Figure 3 and Figure 4 illustrate the effects of the fluid viscosity coefficient on the vibration of a mass in the solid-phase and liquid-phase wave fields, respectively.

Figure 1 and Figure 2 show the presence of four types of waves in the two-phasic porous material, namely, fast quasi-P1 wave, fast quasi-S1 wave, slow quasi-S2 wave, and slow quasi-P wave from outside to inside, which are the same as the conventional Biot and BISQ models. This also proves the correctness of the proposed method in this paper. An analysis of the figures shows that the slow quasi-P wave in the solid phase is weaker than in the liquid phase. Even when the fluid viscosity coefficient is minimal, the slow quasi-P wave cannot be observed in the solid phase. This is because the slow quasi-P wave is a type of highly dissipative wave. The attenuation is faster in the solid phase because solids are more rigid than liquids. With the increase of the viscosity coefficient, the fast quasi-P wave and the fast quasi-S1 wave in the solid and liquid phase all weaken, but the slow quasi-P wave and the slow quasi-S2 all enhance. As the fluid viscosity coefficient increases, the coupling between the solid and liquid phases is enhanced. Therefore, both the fast quasi-P1 wave and the fast quasi-S1 wave all weaken in the source loading direction. However, in the direction of vertical source loading, the slow quasi-S2 wave and the slow quasi-P wave all enhance due to certain total energy. Due to the enhanced coupling between the solid and liquid phases, the slow quasi-P wave in the solid phase enhances in the presence of the enhanced slow quasi-P wave in the liquid phase. In addition, the transverse waves can be seen in the liquid phase does not mean that the transverse waves can propagate in the liquid phase, but rather because the vibrations in the solid and liquid phases are coupled together.

In Figure 3 and Figure 4, we can see that as the viscosity coefficient increases, the amplitudes of both the fast quasi-P wave and the fast quasi-S wave in the solid phase decrease and the phase is delayed. While the fast quasi-P wave amplitude in the liquid phase diminishes, the quasi-S wave amplitude increases, and the phase is delayed. For the solid phase, as the viscosity coefficient increases, the relative motion between the solid and liquid becomes weaker, and the coupling effect between the liquid phase and the solid phase becomes stronger. Therefore, the solid phase energy becomes weaker, and the phase is delayed. As the viscosity coefficient increases, the solid-phase fast quasi-P wave and the liquid phase fast quasi-P wave couple each other, resulting in the weakening of both. At certain total energy, the residual energy in the quasi-S2 wave in the liquid phase enhances in the direction of vertical source loading. Therefore, the quasi-S wave amplitude increases. The above three models are discussed under a certain defined solid skeleton quality factor. Of course, we can also discuss them under different quality factors based on our proposed new fractional-order BISQ model.

### 3.2. Single Layer Models with Different Quality Factor Groups

Elastic wave generates pressure in the solid skeleton, and the pressure induces fluid flow relative to the solid skeleton in a two-phase porous material. The relative flow is known as wave-induced flow. Small particles in the fluid will slowly block the pore space. This process has a relaxation property that can lead to elastic wave attenuation. In order to analyse the influence of the attenuation on elastic wave propagation in viscoelastic two-phase VTI porous materials in detail, the single-layer models with different quality factor groups are designed. Some basic parameters are shown in Table 2.

**Figure 5:**
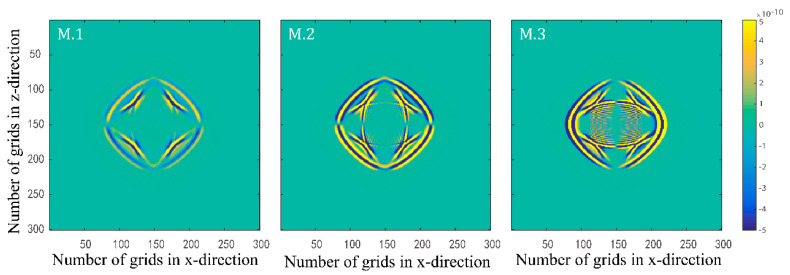
Snapshots of solid–phase z–direction velocity components with three different quality factor groups at t=0.15 s.

**Figure 6:**
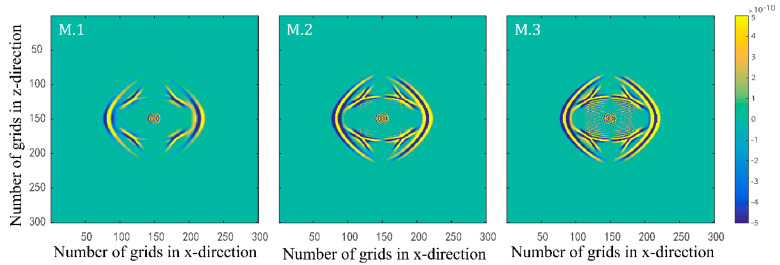
Snapshots of liquid phase x–direction velocity components with three different quality factor groups at t=0.15 s.

**Figure 7:**
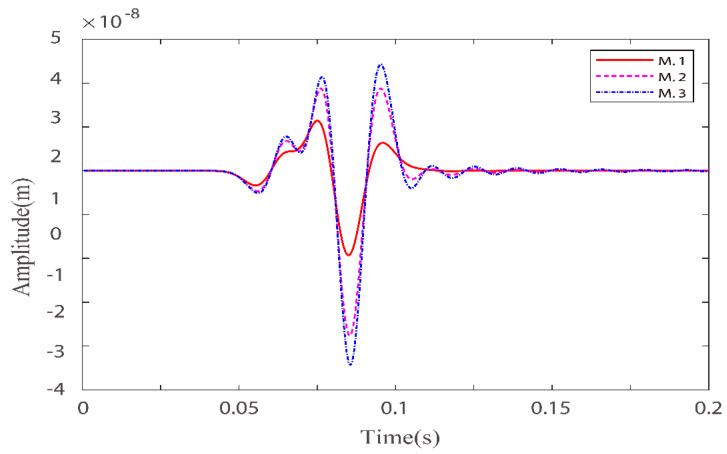
Comparison of solid-phase z–direction velocity components with three different quality factor groups at point (160,160).

**Figure 8:**
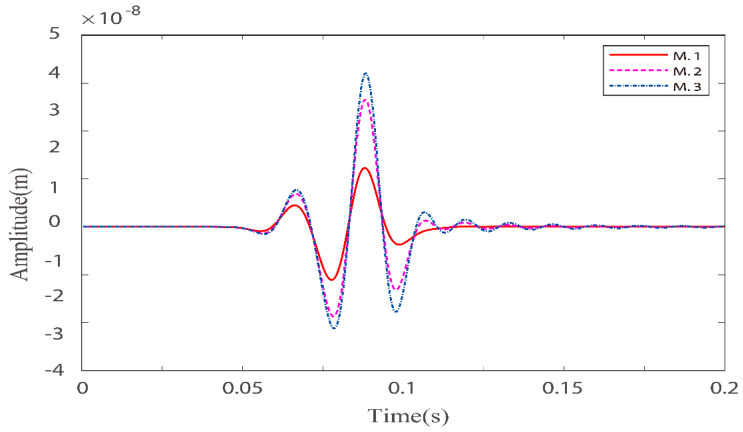
Comparison of liquid–phase x–direction velocity components with three different quality factor groups at point (160,160).

Numerical simulations are performed for the three models in Table 2. Figure 5 and Figure 6 illustrate the effects of quality factors on the solid-phase and liquid-phase wave fields, respectively. Figure 7 and Figure 8 illustrate the effects of the fluid viscosity coefficient on the vibration of a mass in the solid-phase and liquid-phase wave fields, respectively.

In Figure 5 and Figure 6, the amplitudes of the waves all enhance as the quality factors increase. As the quality factors increase, the viscoelasticity of the solid skeleton becomes weaker, and the attenuation of elastic waves becomes weaker. As a result, the amplitudes of the waves enhance in both solid and liquid phases. One of the most important features of the new model proposed in this paper is the freedom to set the viscoelasticity of the solid skeleton to perform numerical simulations of the wave field in a two-phase porous material.

In Figure 7 and Figure 8, for quasi-p wave and quasi-s wave, the mass vibration phase is delayed as the quality factors increase. This is because as the viscoelasticity of the solid skeleton becomes weaker, the rigidity becomes stronger. Masses can reach higher peaks, and reaching higher peaks takes longer. The result is the all delay phases.

### 3.3. Double Layer Model with Buried Body

If the viscoelastic two-phasic porous material is double-layered with a buried body in it, the propagation of the elastic wave will be more complicated. Therefore, we design a double-layer model containing a rectangular buried body to study the elastic wave scattering characteristics of the buried body. Some basic parameters are shown in Table 3.

**Figure 9:**
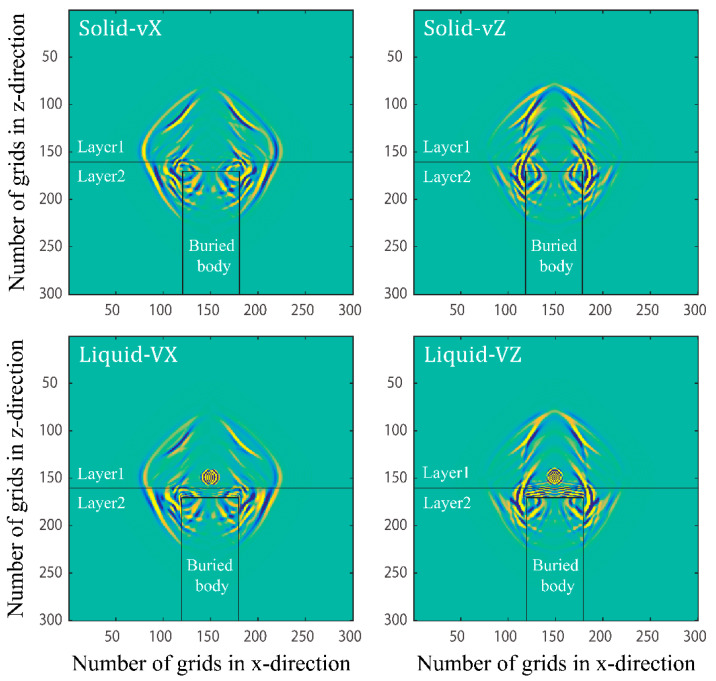
Snapshots of the double layers model with buried body at t=0.15 s.

In Figure 9, the location of the buried body and the snapshots of the wave field in the double-layer containing the buried body are given.

Fast quasi-P, fast quasi-S1, slow quasi-S1, and slow quasi-P waves are reflected, transmitted, and converted at the stratigraphic interface. Multiple types of waves can be observed simultaneously in the wavefield snapshot, and the wavefield information is rich. In addition to this, due to the difference in the quality factors of the upper and lower layers of the solid phase, the difference in the upper and lower amplitudes of each wave can be clearly observed. There are two reflected waves in the upper layer of the stratigraphic interface and two transmitted waves in the lower layer, which come from the stratigraphic interface and the upper interface of the buried body, respectively. Complex reflection, transmission, and conversion phenomena also occur at the corners of the buried body. These waves superimpose with the transmitted and converted waves at the stratigraphic interface, which produces a more complex wave field. The study of transmission and scattering mechanisms of elastic waves in two-phasic porous materials containing buried bodies is important for analysing the stress distribution of the buried bodies in two-phasic porous materials in the elastic wave field. For example, it can be used to analyse the force of underground buildings in seismic wave fields.

## 4. Conclusions

In this paper, the constant Q fractional-order theory is combined with the BISQ theory to establish a more generalised constant Q fractional-order wave model to describe the propagation of elastic waves in the viscoelastic two-phasic porous materials. The two-dimensional velocity-stress wave propagation equation is derived. Fractional-order time derivatives are truncated by the short-time memory method. Numerical simulations of wave fields in the material for different cases are performed. By analysing the simulation results, we can draw several conclusions.

As the fluid viscosity coefficient increases, the pore fluid becomes more viscous. The fluid flow between the pores and fractures becomes slower, hindering the P-wave propagation. The fast quasi-P and quasi-S1 waves in the solid phase and the fast quasi-P wave energy in the liquid phase weaken. Under the condition of constant total energy and weak dissipation of relative motion, the residual energy of the slow quasi-S2 wave in the liquid phase is enhanced. As the quality factors increase, the viscoelasticity of the solid skeleton of the viscoelastic porous material decreases and the rigidity becomes stronger. The amplitudes of the waves all enhance. The time required to reach higher peaks becomes longer, resulting in the phase delay.

In a double-layer material containing buried body, complex scattering, transmission, and waveform conversion phenomena occur at the stratigraphic interface, buried body interface, and corners. In-depth study of wave scattering, transmission, and conversion phenomena in viscoelastic two-phasic porous materials or complex buried structures and analysis of various wave propagation characteristics can deepen the understanding of wave propagation mechanism in actual viscoelastic two-phasic porous materials. This may help conduct more in-depth research on viscoelastic two-phasic porous materials using multiwave elastic data, with important theoretical and practical applications.

This paper also has several shortcomings. Physical experiments should be carried out to further verify the accuracy of the model developed. The BISQ model assumes that the pore fluid flow satisfies Darcy’s law. In reality, the fluid may not satisfy Darcy’s law due to the semiconnectivity of the pores or the presence of particles in the fluid that can block the pores. Therefore, a non-Darcy’s law BISQ model should be considered.

## Figures and Tables

**Table 1:** Three models with different viscosity coefficients.

**Solid Phase Parameters**	**Unit**	**Model 1**	**Model 2**	**Model 3**
$\rho_{s}$	$\mathrm{kg}/m^{3}$	2050	2050	2050
$K_{s}$	Gpa	7.78	7.78	7.78
ϕ	-	0.3	0.3	0.3
$Q_{p}$	-	30	30	30
$Q_{s}$	-	15	15	15
$v_{p∥}$	m/s	3300	3300	3300
$v_{p⊥}$	m/s	3167	3167	3167
$v_{s}$	m/s	1600	1600	1600
**Fluid Phase Parameters**	**Unit**	**Model 1**	**Model 2**	**Model 3**
$\rho_{f}$	$\mathrm{kg}/m^{3}$	1040	1040	1040
$K_{f}$	Gpa	0.372	0.372	0.372
$\rho_{a}$	$\mathrm{kg}/m^{3}$	420	420	420
k	md	20	20	20
η	cp	0.001	0.05	0.09

**Table 2:** Three models with three different quality factor groups.

**Solid Phase Parameters**	**Unit**	**Model 1**	**Model 2**	**Model 3**
$\rho_{s}$	$\mathrm{kg}/m^{3}$	2050	2050	2050
$K_{s}$	Gpa	7.78	7.78	7.78
ϕ	-	0.3	0.3	0.3
$Q_{p}$	-	10	30	60
$Q_{s}$	-	5	15	30
$v_{p∥}$	m/s	3300	3300	3300
$v_{p⊥}$	m/s	3167	3167	3167
$v_{s}$	m/s	1600	1600	1600
**Fluid Phase Parameters**	**Unit**	**Model 1**	**Model 2**	**Model 3**
$\rho_{f}$	$\mathrm{kg}/m^{3}$	1040	1040	1040
$K_{f}$	Gpa	0.372	0.372	0.372
$\rho_{a}$	$\mathrm{kg}/m^{3}$	420	420	420
k	md	20	20	20
η	cp	0.001	0.001	0.001

**Table 3:** Double-layer model with three different quality factor groups.

**Solid Phase Parameters**	**Unit**	**Layer 1**	**Layer 2**	**Buried Body**
$\rho_{s}$	$\mathrm{kg}/m^{3}$	2050	2050	2050
$K_{s}$	Gpa	7.78	7.78	7.78
ϕ	-	0.3	0.3	0.3
$Q_{p}$	-	20	30	40
$Q_{s}$	-	10	15	20
$v_{p∥}$	m/s	3300	3300	3300
$v_{p⊥}$	m/s	3167	3167	3167
$v_{s}$	m/s	1600	1600	1600
**Fluid Phase Parameters**	**Unit**	**Layer 1**	**Layer 2**	**Buried Body**
$\rho_{f}$	$\mathrm{kg}/m^{3}$	1040	1040	1040
$K_{f}$	Gpa	0.372	0.372	0.372
$\rho_{a}$	$\mathrm{kg}/m^{3}$	420	420	420
k	md	20	20	20
η	cp	0.01	0.01	0.01
